# An analysis of the psychometric properties of the medication safety competence scale in Turkish

**DOI:** 10.1186/s12912-024-02240-0

**Published:** 2024-08-21

**Authors:** Ayşe Aydinli, Kamuran Cerit

**Affiliations:** 1 Department of Fundamental Nursing, Faculty of Health Sciences, Suleyman Demirel University, Isparta, Turkey; 2 Department of Nursing Management, Faculty of Health Sciences, Suleyman Demirel University, Isparta, Turkey

**Keywords:** Nurses, Medication, Patient safety, Clinical competence, Reliability and validity

## Abstract

**Purpose:**

Considering the key roles and responsibilities of nurses in ensuring medication safety, it is necessary to understand nurses’ competence in medication safety. Therefore, it was aimed to introduce a scale evaluating the medication safety competence of nurses into Turkish and to contribute to the literature by determining the medication safety competence levels of nurses.

**Methods:**

A methodological and descriptive research design was utilised. The population consisted of nurses in Turkey, and the sample comprised 523 nurses who volunteered to participate.

**Results:**

The content validity index of the scale was 0.98, and the scale showed a good fit (χ^2^/df = 3.00, RMSEA = 0.062). The Cronbach’s alpha coefficient of the scale was 0.97, indicating high reliability. The mean score was 4.12, which was considered high. Participants who were 40 years old or above, married, and graduates of health vocational schools or postgraduate programs, along with those who had received medication safety training, had higher medication safety competence scores.

**Conclusion:**

This study presents strong evidence that the Turkish version of the Medication Safety Competency Scale is valid and reliable when administered to nurses. The participants in this study had high levels of medication safety competence.

**Supplementary Information:**

The online version contains supplementary material available at 10.1186/s12912-024-02240-0.

## Introduction

A medication error is defined as a preventable event at any stage of drug therapy that results in incorrect drug use or harms the patient [1]. According to World Health Organization (WHO) reports, medication errors account for 20% of medical errors [2]. Medication errors decrease patients’ quality of life and result in high costs for healthcare institutions [3]. They can also lead to complications, permanent disability, and death [1]. Globally, concerns about medication errors are increasing, and various reports have emphasised the importance of reducing medication errors and improving patient safety [2, 4, 5].

Patient safety involves ensuring that patients are not harmed while receiving care, and medication safety is among the most important elements of patient safety [6]. Medication safety can be defined as ensuring that medications have the maximum therapeutic effect while minimising and preventing adverse reactions and accidental injuries during medication use [4]. Medication safety is a multidisciplinary and multi-stage process. Nurses constitute the majority of the healthcare team and are involved in many stages of the medication administration process; they are at the centre of medication administration and are involved in the most critical stage when any potential errors reach the patient [7].

Traditional nursing curricula consider the “right principles” as a basic standard for safe medication practices. However, nurses’ role in ensuring medication safety encompasses many other principles [3]. Limiting nurses’ responsibilities regarding medication safety to the right principles does not address all aspects of errors [8]. Medication safety requires nurses to use clinical judgment before, during, and after interventions. Nurses’ experience and knowledge are integral components of safe medication management in nursing practice [9]. Adverse effects caused by improper prescription, administration, or monitoring of medications can be decreased through good nursing practice [8].

Considering the high prevalence of medication errors and the key role of nurses in ensuring medication safety, the medication safety competence of nurses must be determined [4]. However, very few studies have assessed nurses’ medication safety competence [3–6]. Moreover, a Turkish scale to assess nurses’ the of and attitudes towards medication safety competence is needed.

Therefore, this study introduced a Turkish version of a scale evaluating the medication safety competence of nurses and administered it to nurses, contributing to the literature by determining the medication safety competence levels of nurses.

## Methods

This study adapted the Medication Safety Competence Scale (MSCS) developed by Park and Seomun (2021) for use in a Turkish context and assessed its validity and reliability; subsequently, the scale was used to determine nurses’ the of medication safety competence [5]. Differences in the of medication safety competence between nurses with varying demographic characteristics were also investigated.

### Study design and participants

This study was conducted methodologically and descriptively. This study was structured and reported according to the Strengthening the Reporting of Observational Studies in Epidemiology (STROBE) checklist [10].

The population of the study comprised nurses working at various hospitals in Turkey. Convenience sampling method was used. The sample consisted of 523 nurses who were native Turkish speakers and voluntarily participated in the study. No one refused to participate in the study. There were no missing data except for 36 data related only to the worked clinic was conducted. However, these 36 data were not excluded; instead, analyses related to the clinic variable were conducted using 487 data (Tables 1 and 2).

**Table 1:** The distribution of demographic characteristics of participants

Socio-Demographic Characteristics		*N*	%
**Gender**	Female	494	94.5
	Male	29	5.5
**Age**	20–29 years	320	61.2
	30–39 years	144	27.5
	40 years and above	59	11.3
**Marital status**	Married	281	53.7
	Single	242	46.3
**Education status**	Health Vocational High School (HVHS)	34	6.5
	Associate degree	40	7.6
	Bachelor’s degree	380	72.7
	Postgraduate	69	13.2
**Professional experience**	1–5 Years	287	54.9
	6–10 Years	100	19.1
	11–15 Years	68	13.0
	16 Years and above	68	13.0
**Hospital Type**	Research and university	183	35.0
	Government	180	34.4
	City	83	15.9
	Private and other	77	14.7
**Clinic (N: 487)**	Internal units	132	27.1
	Surgical units	91	18.7
	Intensive care	124	25.5
	Emergency	52	10.7
	Pediatrics and obstetrics	53	10.9
	Privileged units*	35	7.2
**Have you received training or courses on medication safety?**	Yes	414	79.2
	No	109	20.8
**Do you think that the principles of medication administration are followed in the clinic where you work?**	Yes	416	79.5
	No	107	20.5
**Do you think medication administration in the clinic where you work is carried out according to the hospital’s medication administration rules and procedures?**	Yes	401	76.7
	No	122	23.3

*Endoscopy, chemotherapy, management, etc.

**Table 2:** Comparison of participants’ of medication safety competence according to demographic characteristics

Demographic Characteristics	Groups	*N*	m ± sd	Mean rank	Test Value	*P*	Difference
Gender*	Female	494	4.11 ± 0.59	264.11	Z= -1.321	0.187	Ø
	Male	29	3.98 ± 0.57	225.98			
Age**	20–29 years	320	4.07 ± 0.55	248.80	χ^2^= 7.991	**0.018**	40 and over > 20–29 years
	30–39 years	144	4.15 ± 0.61	273.94			
	40 years and above	59	4.20 ± 0.72	304.42			
Marital Status*	Single	281	4.06 ± 0.52	243.75	Z= -2.976	**0.003**	Married > Single
	Married	242	4.15 ± 0.66	283.19			
Education Level**	HVHS	34	4.35 ± 0.40	328.43	χ^2^=18.332	**0.000**	HVHS > Bachelor’s degree Postgraduate > Bachelor’s degree
	Associate degree	40	4.18 ± 0.56	287.10			
	Bachelor’s degree	380	4.05 ± 0.58	245.31			
	Postgraduate	69	4.26 ± 0.68	306.62			
Professional experience **	1–5 Years	287	4.02 ± 0.54	236.55	χ^2^ = 20.932	**0.000**	6–10 years = 11–15 years = 16 years and above > 1–5 years
	6–10 Years	100	4.16 ± 0.56	276.18			
	11–15 Years	68	4.22 ± 0.65	294.16			
	16 Years and above	68	4.25 ± 0.71	316.42			
Hospital Type **	Research and university	183	4.14 ± 0.54	264.29	χ^2^= 11.303	**0.010**	Private and other > Govern.
	Government	180	4.00 ± 0.64	236.39			
	City Private and other	83 77	4.16 ± 0.50 4.23 ± 0.65	275.14 302.24			
Clinic (*n* = 487) **	Internal units	132	4.15 ± 0.49	252.19	χ^2^= 8.261	0.142	Ø
	Surgical units	91	4.09 ± 0.60	232.19			
	Intensive care	124	4.12 ± 0.53	241.87			
	Emergency	52	3.97 ± 0.71	212.58			
	Pediatrics and obstetrics	53	4.25 ± 0.69	284.75			
	Privileged units*	35	4.02 ± 0.67	236.34			
Have you received training or courses on medication safety?	Yes	414	4.15 ± 0.57	273.55	Z= -3.406	**0.001**	Yes > No
	No	109	3.93 ± 0.64	218.15			
Do you think that the principles of medication administration are followed in the clinic where you work?	Yes	416	4.17 ± 0.59	280.41	Z= -5.496	**0.000**	Yes > No
	No	107	3.86 ± 0.54	190.41			
Do you think that medication administration in the clinic where you work is carried out in accordance with the hospital’s medication administration rules and procedures?	Yes	401	4.17 ± 0.57	278.84	Z= -4.620	**0.000**	Yes > No
	No	122	3.91 ± 0.61	206.66			

Note: m Mean, sd Standard Deviation, * Mann Whitney U Test **Kruskal Wallis Test

The sample size for conducting CFA was determined to be at least 10 times the number of scale items [11]. This rule was met for 36 items with 523 cases. In descriptive analyses, the sufficiency of the sample size was determined by a post hoc power analysis. As a result of the post hoc power analysis conducted with G*Power 3.1.9.7, the power of the study was calculated as 85% with an effect size of 0.26 and a significance level of 0.05 [12].

### Data collection

A demographic information form was used to determine the demographic characteristics of the nurses, and the MSCS was used to determine their the of medication safety competence.

The demographic information form consists of 10 questions. The form, which was prepared by the researchers in line with the literature, consists of questions with demographic characteristics of the nurses such as age, gender and worked clinics [3–6].

The MSCS was developed by Park and Seomun in 2021 [5]. The scale consists of 36 items divided into six subdimensions. The dimensions are patient-centred medication management (Items 1,4,5, 6,7,8, 13,24,26); multidisciplinary collaboration (Items 20,27,30,33); safety risk management (Items 2,15,16,21,25,28); management of effecting factors (Items 3,9,11,12,14,18); improvement of safety problems (Items 10,19,22,29,31,32,34,35); and responsibility in the nursing profession (Items 17,23,36). The scale is a five-point Likert scale. The total score ranges from 36 to 180. Scores between 36 and 75 represent poor medication safety competence, scores between 76 and 130 indicate moderate medication safety competence, and a score of 180 represents high medication safety competence [6].

Data were collected through an online survey between February 1 and March 31, 2023. Participants were reached via social media (WhatsApp, Instagram story, etc.) and invited to participate in the online survey prepared through Google Forms. An informed consent form was attached to the first part of the questionnaire and participation was voluntary. The response time of the questionnaires was 8–10 min.

### Procedure

This study was conducted in two stages: methodological and descriptive.

#### Methodological stage

This stage consisted of translation and psychometric testing. To adapt the scale for use in a Turkish context, permission was obtained from the researchers who developed the original scale. Back translation was used for language validity. The content validity of the Turkish version of the scale was tested. After receiving expert opinions, the scale was translated back into English (supplementary file-1). After the translated version was sent to the researchers who developed the scale and approval was obtained, data collection was started with the Turkish form. Data were then collected for psychometric testing. The validity and reliability of the original scale were tested with data from the 523 participants.

#### Descriptive stage

In this stage, nurses’ the of medication safety competence were determined and analysed according to several demographic characteristics. Data from the 523 participants were used in this stage.

### Data Analysis

Data were analysed with the Statistical Package for the Social Sciences (SPSS) v. 26.0, LISREL v. 8.80, and Microsoft Excel.

#### Descriptive statistics

Means and standard deviations were calculated for continuous data, and percentages were calculated for categorical data. The adequacy of the multivariate normal distribution of the data was assessed using Mardia’s skewness and kurtosis tests. Mann–Whitney U and Kruskal–Wallis tests were used for comparison between groups of nurses with different demographic characteristics.

#### Item analysis

To determine whether the scale had an ideal discrimination ability, the total score of the scale was ranked from high to low, and the difference between the first 27% and the last 27% was analysed. Furthermore, item-total score correlation coefficients were calculated.

#### Validity analysis

Eleven experts rated the items of the adapted scale from 1 to 4 for content validity (1: not appropriate; 2: partially appropriate, the item needs to be revised; 3: appropriate but minor changes are needed; 4 very appropriate). The item content validity index (I-CVI) and the scale content validity index (S-CVI) were calculated using the method proposed by Davis (1992) [13]. The I-CVI is the ratio of the number of experts who assign each item 3 or 4 points to the total number of experts. The S-CVI is the average I-CVI for all items.

Confirmatory factor analysis (CFA) was applied for the construct validity, and fit indices were evaluated. The values of chi-square (χ^2^)/degree of freedom (df), comparative fit index (CFI), root-mean-square error of approximation (RMSEA), non-normed fit index (NNFI), normed fit index (NFI), standardised root mean square residual (SRMR), root mean square residual (RMR), goodness of fit index (GFI), and adjusted goodness of fit index (AGFI) were examined. Also average variance extracted (AVE) and Construct reliability (CR) were examined for convergent validity.

#### Reliability analysis

Cronbach’s alpha (α) and split-half reliability were calculated to assess internal consistency. To assess test–retest reliability, intra-class correlation (ICC) was calculated by collecting data from 30 nurses at 2-week intervals. The data obtained for the test-retest were not included in the sample.

### Ethical considerations

#### Ethical approval

of the study was obtained from the Suleyman Demirel University Institutional Ethics Committee (decision number: 87432956-050.99-423263). Informed consent was obtained from the participating nurses in the first part of the online survey. The study was carried out in accordance with the principles of the Declaration of Helsinki.

## Results

Most participants (94.5%) were female, 61.2% were between the ages of 20–29 years, 53.7% were married, 72.7% had a Bachelor’s degree, and 54.9% had 1–5 years of professional experience. Furthermore, 79.2% of the participants reported that they had received training or courses on medication safety, 79.5% reported that medication administration principles were followed in the clinic where they worked, and 76.7% stated that medication administration was performed following the hospital’s medication administration rules and procedures (Table 1). The results of the methodological and descriptive stages of the study are provided in the following two sections.

### Results of the methodological stage

The scale was translated into Turkish by four translators who were native Turkish speakers and fluent in English. The researchers then combined the four translations into a single form. In the second step, this form was translated back into English by an expert who was not one of the previous translators.

The expert review was conducted by nine nursing instructors and two nurses with master’s degrees. The I-CVI ranged from 0.80 to 1.00, and the S-CVI was 0.98. To assess the face validity of the Turkish form, a preliminary application was performed with 20 nurses. To ensure that they were comprehensible in Turkish, the expression “human factors” in the item “Understanding the role of human factors, such as fatigue, that affect medication safety” was changed to “personal factors”, and the expression “understanding the role” in “Understanding the role of environmental factors such as workflow, ergonomics, and resources, which affect medication safety” was changed to “understanding the effect”. With these adjustments, the scale form adapted to Turkish was finalised. Data from the pilot study were not included in the sample.

**Fig. 1:**
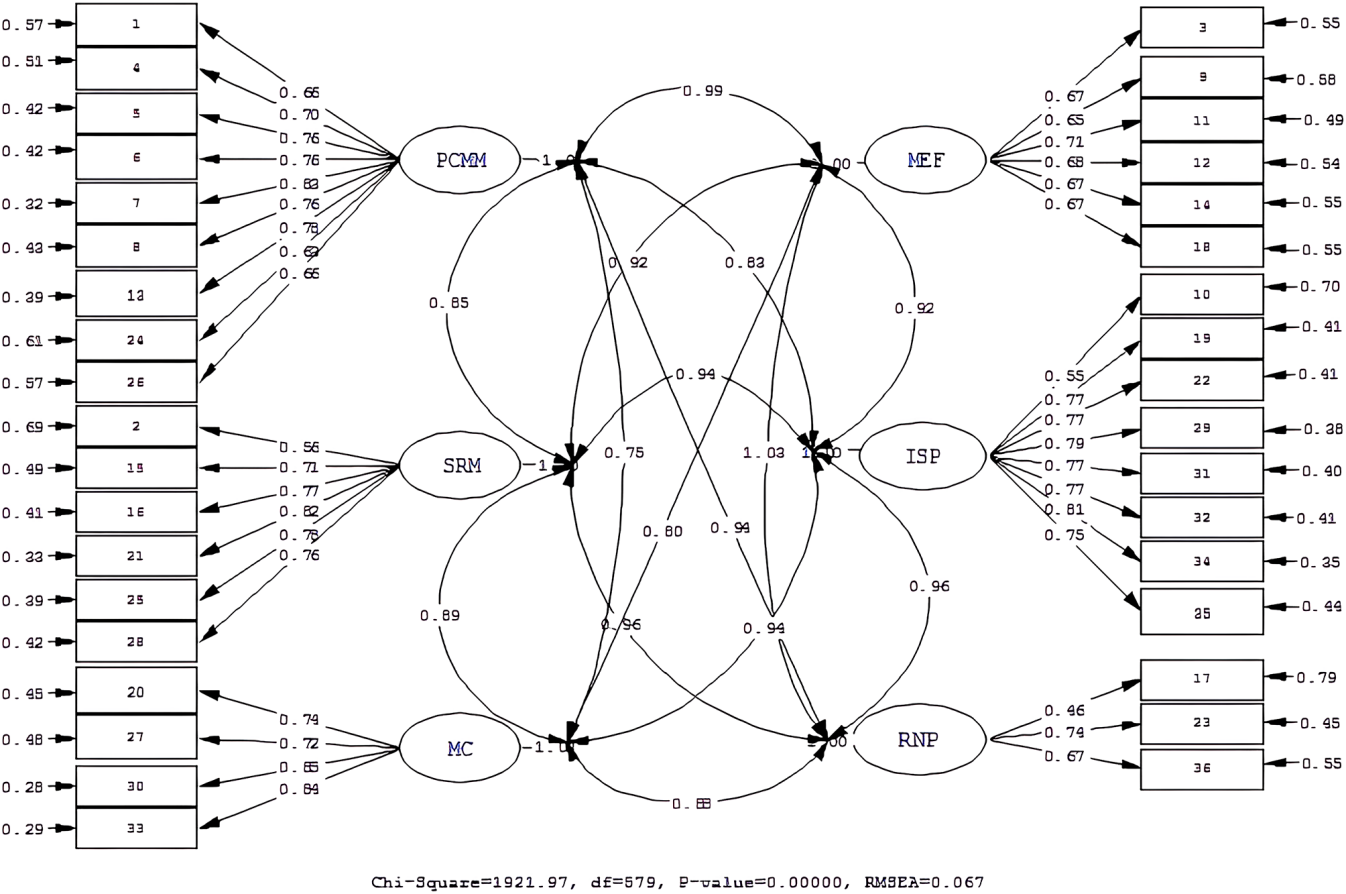
CFA results of Model I: standard loadings and error variances

**Fig. 2:**
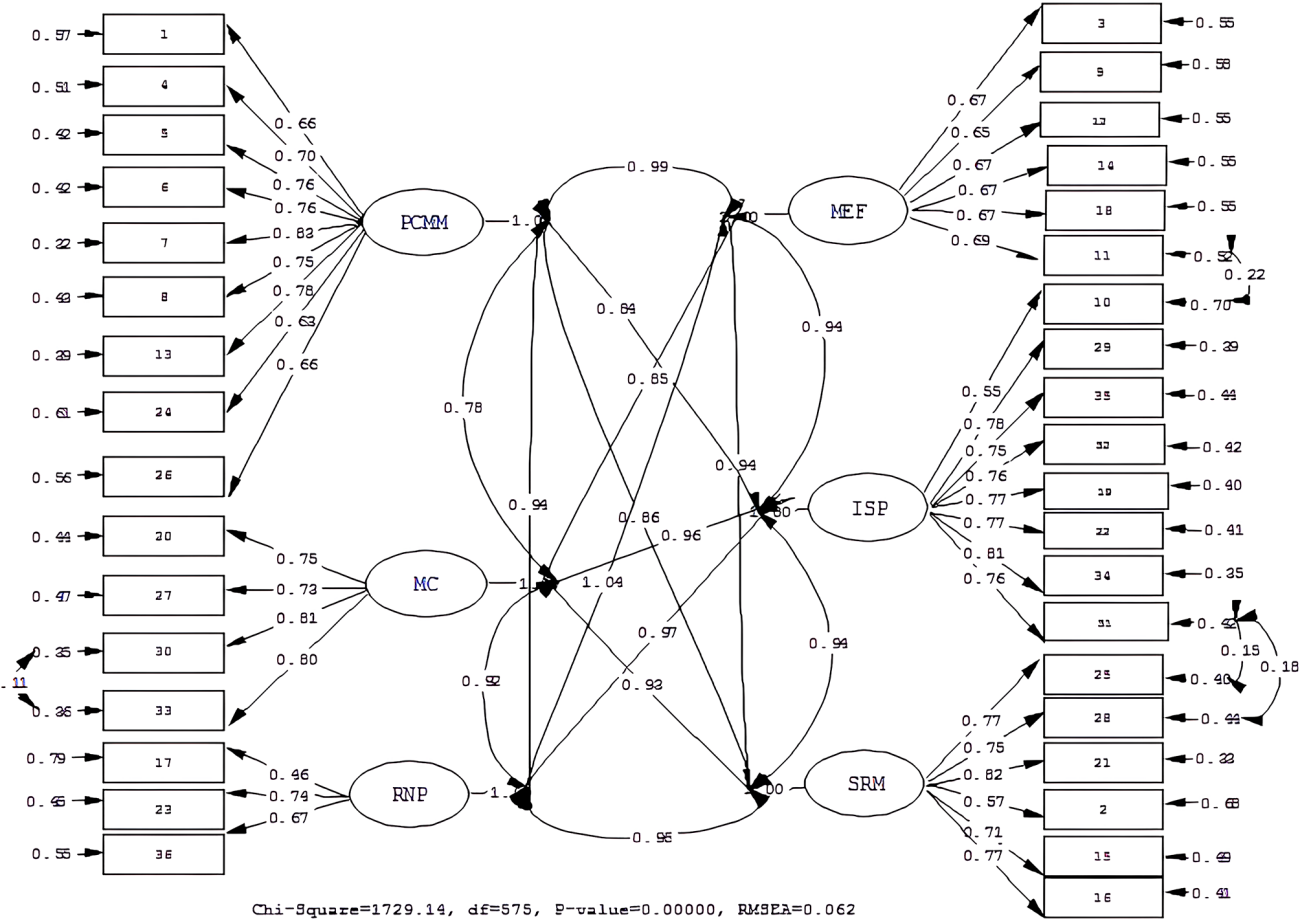
CFA results of Model II: standard loadings and error variances

The results of CFA are shown in Table 3. The fitness indices of the original scale (model 1) (χ^2^/df = 1921.97/579 = 3.32, RMSEA = 0.067, CFI = 0.98) were determined to be at an acceptable level (Fig. 1). However, modification indices were examined, and the original scale was modified sequentially as follows: item 28 and item 31, item 25 and item 31, item 10 and item 11, respectively. The modification of model 2 was achieved by freeing the error terms (permitting correlated errors) of the items without excluding any items (Fig. 2).

**Table 3:** CFA results of Model I and Model II: fit indices

Indices	Perfect Fit Criterion	Acceptable Fit Criterion	Results of Model 1	Results of Model 2
χ^2^/df (p)	0–3	3–5	1921.97/579: 3.32 (*p* = 0.00)	1729.14 /575: 3.00 (*p* = 0.00)
RMSEA	0.00 ≤ RMSEA ≤ 0.05	0.05 ≤ RMSEA ≤ 0.10	0.067	0.062
CFI	0.95 ≤ CFI ≤ 1.00	0.90 ≤ CFI ≤ 0.95	0.098	0.99
NFI	0.95 ≤ NFI ≤ 1.00	0.90 ≤ NFI ≤ 0.95	0.98	0.98
NNFI	0.95 ≤ NNFI ≤ 1.00	0.90 ≤ NNFI ≤ 0.95	0.098	0.98
RMR	0.00 ≤ RMR ≤ 0.05	0.05 ≤ RMR ≤ 0.08	0.045	0.042
SRMR	0.00 ≤ SRMR ≤ 0.05	0.05 ≤ SRMR ≤ 0.08	0.060	0.057
GFI	0.95 ≤ GFI ≤ 1.00	0.90 ≤ GFI ≤ 0.95	0.76	0.78
AGFI	0.90 ≤ AGFI ≤ 1.00	0.85 ≤ AGFI ≤ 0.90	0.72	0.74

The fitness indices of the final scale (model 2) were as follows: χ^2^/df = 3.00, RMSEA = 0.062, CFI = 0.99, NFI = 0.98, GFI = 0.78, AGFI = 0.74. The standard loadings of the items in the final scale ranged from 0.46 to 0.83. The squared multiple correlations (SMC-R^2^) ranged from 0.21 to 0.68 (Table 4). Furthermore, the fit indices attained in both models were acceptable. Nevertheless, model 2 exhibited superior χ2/df and RMSEA values, indicating that it outperformed model 1.

**Table 4:** CFA and item analysis results of the Model II

Factor	Item	Parameter significance estimation		Factor Reliability	Item Analysis
PCMM		Std. Estimate	Unstd. Estimate	*SMC (*R*^2^)	Item total score correlation
	1- Planning care in the medication process…	0.66	0.49	0.43	0.606
	4- Detecting adverse reactions in medication….	0.70	0.59	0.49	0.663
	5- Giving confidence to patients and caregivers in the medication process….	0.76	0.55	0.58	0.659
	6- Giving a sense of stability through clear and consistent communication with patient…	0.76	0.52	0.58	0.653
	7- Evaluating my nursing practice in the medication process…	0.83	0.57	0.68	0.735
	8- Documentation of assessment, planning, administration of medication, and evaluation of outcomes….	0.75	0.55	0.57	0.688
	13- Communicating individually according to patients’ condition and level in the medication process……	0.78	0.54	0.61	0.711
	24- Practicing medication care with responsibility for the safety of patients…	0.63	0.48	0.39	0.599
	26- Effective patient training to help patients speak of the symptoms of adverse effects…	0.66	0.62	0.44	0.716
**ISP**	10- Having a questioning attitude and speaking up when you see something that may be unsafe….	0.55	0.41	0.30	0.583
	19- Establish prevention measures when medication errors or near-misses occur….	0.77	0.63	0.60	0.755
	22- Trying to create a supportive environment that encourages people to talk about problems when medication errors or near-misses occur….	0.77	0.69	0.59	0.746
	29- Improving the complex and vulnerable way of medication safety (e.g., incorrect administration practices)…	0.78	0.63	0.61	0.738
	31- Reporting to a nursing manager or supervisor when medication errors or near-misses occur….	0.76	0.75	0.58	0.728
	32- Analyzing the case to find the root cause of the medication error……	0.76	0.68	0.58	0.702
	34- Establishing prevention measures when adverse drug events occur…	0.81	0.63	0.65	0.769
	35- Identifying the root cause rather than blaming the individual when medication errors or near-misses occur….	0.75	0.60	0.56	0.694
**MEF**	3- Administration according to the right way (patient, drug, dose, route, and time)…	0.67	0.43	0.45	0.631
	9-Understanding the role of human factors, such as fatigue, that affect medication safety…	0.65	0.50	0.42	0.619
	11-Describing prevention activities for medication safety……	0.69	0.52	0.48	0.693
	12- Finding information about medication from different sources (e.g., drug information management system, hospital pharmacies, literature information, etc.) ……	0.67	0.56	0.45	0.652
	14- Using information technology and computerized systems (e.g., barcodes, electronic medical records) for medication safety……	0.67	0.56	0.45	0.656
	18- Understanding the role of environmental factors such as workflow, ergonomics, and resources, which effect medication safety….	0.67	0.60	0.45	0.684
**SRM**	2-Assess the need for medication by checking patients’ condition and examination results prior to administration…	0.57	0.47	0.32	0.581
	15-Managing the medicine according to the hospital’s medication management guidelines (e.g., high-risk medication guidelines) ….	0.71	0.61	0.51	0.709
	16- Coping quickly according to hospital protocol when adverse drug events occur……	0.77	0.70	0.59	0.732
	21- Coping quickly according to hospital protocol when medication errors or near-misses occur……	0.82	0.77	0.67	0.770
	25- Reporting the adverse drug events according to the reporting system…	0.77	0.80	0.60	0.731
	28- Reporting to a nursing manager or supervisor when medication errors or near-misses occur…	0.75	0.73	0.56	0.712
**MC**	20- Communicating effectively between multidisciplinary members to address medication safety issues…	0.75	0.71	0.56	0.698
	27- Collaborating with other departments (e.g., medicine department, laboratory, another ward, etc.) for medication safety…	0.73	0.71	0.53	0.671
	30-Sharing decision-making between multidisciplinary to address medication safety issues….	0.81	0.70	0.65	0.742
	33- Collaborating with multidisciplinary professionals to address medication safety issues…	0.80	0.73	0.64	0.728
**RNP**	17- Receiving regular medication safety training….	0.46	0.55	0.21	0.459
	23- Evaluating regularly my knowledge of medication safety……	0.74	0.60	0.55	0.746
	36- Performing medication care with alertness as a professional…	0.67	0.46	0.45	0.647

Note: * Square of Multiple coefficient (R^2^)

For convergent validity, the CRs ranged from 0.68 to 0.79 and were higher than the AVE values (0.40 to 0.60). The correlation between factors ranged from 0.687 to 0.868 (Table 5). Item analyses revealed that the item-total correlations were between 0.46 and 0.77 (Table 4). A statistically significant difference (t: −30.601, *p* < 0.001) was observed between the mean scores of the groups with the lowest 27% of scores and the highest 27% of scores.

**Table 5:** Convergent validity and internal consistency results of the Model II and Mean scores of the participants

Scale and Sub-Scale	m	sd	Correlation coefficients between factors (Discriminant Validity)						Convergent Validity		Internal consistency
			PCMM	ISP	MEF	SRM	MC	RNP	CR	AVE	α
MSCS	**4.11**	**0.59**	**0.914**	**0.941**	**0.916**	**0.932**	**0.868**	**0.812**			0.970
PCMM	4.25	0.57	1.00						0.76	0.53	0.903
ISP	4.07	0.66	0.792	1.00					0.77	0.56	0.907
MEF	4.21	0.59	0.868	0.816	1.00				0.71	0.45	0.831
SRM	4.00	0.73	0.806	0.858	0.813	1.00			0.76	0.54	0.872
MC	3.98	0.78	0.701	0.845	0.700	0.796	1.00		0.79	0.60	0.860
RNP	3.96	0.69	0.687	0.723	0.738	0.731	0.687	1.00	0.68	0.40	0.601

Note: m (mean), sd (standard deviation), CR (Construct Reliability), AVE (Average Variance Extracted), α (Cronbach’s alpha)

As shown in Table 5, the Cronbach’s alpha coefficient of the scale was 0.97. The split-half reliability was 0.912, and the test–retest reliability (ICC) was 0.939.

### Result of the descriptive stage

The participants’ total mean score of MSCS was 147.81 ± 21.29, indicating a average level of medication safety competence. The lowest score was obtained in the RNP dimension (11.88 ± 2.05 points) and the highest score was obtained in the PCMM dimension (38.21 ± 5.14 points) (Table 5). Table 2 presents the comparison of participants’ the of medication safety competence according to their demographic characteristics.

No differences were observed in the participants’ the of medication safety competence according to gender and the type of clinic they worked in. However, significant differences were found according to age, marital status, educational level, professional experience, and the type of hospital in which the nurses worked. Participants aged 40 years and older had higher the of medication safety competence than those aged 20–29 years; married participants had higher than single participants; and those with health vocational school and postgraduate degrees had higher than those with undergraduate degrees. In addition, nurses who received training or courses on medication safety, those who thought that the principles of medication administration were followed in the clinic where they worked, and those who thought that medication administration was performed following the hospital’s medication administration rules and procedures had higher the of medication safety competence (*p* < 0.05).

## Discussion

### Discussion of the Methodological Stage

This study was conducted to determine the validity and reliability of a Turkish version of the MSCS. This study found that the Turkish version of the MSC scale meets the criteria of language validity, content validity, construct validity, and reliability. The validity and reliability of the scale have also been confirmed in Chinese and Persian [4, 6].

In this study, the Turkish version of the scale was created using the back translation method for language validity. Then the content validity of the scale was evaluated according to Davis’s (1992) technique [13]. Because the CVI values of all items were above 0.80, no items were removed at this stage. CFA was performed to verify construct validity. Two models were analysed: the original scale (model 1) and the final scale (model 2). According to the factor loadings and modification indices of model 1 and model 2, the measurement validity of the Turkish version of the MSCS was confirmed. Thus, no items were removed from the scale at this stage.

The χ^2^/df of the final scale was 3.00; this value is considered acceptable, as it is less than 5 [14]. The RMSEA value of 0.062 is an important indicator of the acceptable fit of the final scale. The RMR (0.042) and SRMR (0.057) values showed perfect and acceptable fit, respectively [15]. The CFI was above 0.95, indicating perfect fit [16]. The NFI and NNFI were also above 0.95, indicating perfect fit [17, 18]. In CFA, it is recommended that the factor loadings of the items factors should be above 0.50 [10]. In this study, the factor loadings of the items were between 0.46 and 0.83 (Table 4). Thus, the fit indices and item factor loadings confirmed the construct validity of the final scale. The AVEs of the factors were higher than 0.50 in all subdimensions except for RNP and MEF. Moreover, the CRs were between 0.68 and 0.79, higher than the recommended value of 0.70 and the AVE values (Table 5). These results confirmed the scale’s convergent validity.

Finally, item analyses showed that the items in the scale had good discrimination. Similarly, the Chinese version [4], the original scale [5] and the Persian version [6] also reported high factor loadings, acceptable fit indices and CRs above 0.70. In line with these results, it can be said that the scale provides an adequate level of validity.

### Reliability

The reliability of the scale was evaluated by calculating split-half reliability, Cronbach’s alpha, CR and ICC. Cronbach’s alpha value should be at least 0.70 [11, 19]. In the present study, Cronbach’s alpha was 0.97, whereas it was 0.94 in the Chinese version [4], 0.94 in the original scale [5], and 0.96 in the Persian version [6]. The CR values reported as more appropriate reliability measures for CFA-based studies [11] are higher than the proposed value of 0.70. These results show that the scale has good internal reliability in samples from different cultures. In this study, the split-half reliability of the scale was 0.912, and the test–retest reliability was 0.939. The split-half reliability of the Chinese version of the scale was 0.671, and the test–retest reliability was 0.703 [4]. For the Persian version, the test–retest reliability was also 0.90 [6]. These values indicate the stability of the various versions of the scale [20].

### Discussion of the descriptive phase

To date, nurses’ medication safety competency has generally been examined within the framework of the right principles [21, 22] or reporting medication errors [23, 24]. However, medication safety is a concept that transcends the right principles [3, 8], and previous studies measuring nurses’ medication safety competency have been insufficient. The medication safety competence scores of the nurses in this study were average. Mohebi et al. (2024) also reported the medication safety competence of nursing students at an average level. These results could indicate that the medication safety competence of nurses and students were adequate but needed further development [25]. These results may be explained by the fact that most of the nurses in the study received training or courses on medication safety. Moreover, in recent years, in Turkey and other countries, patient and medication safety issues have been important issues of health policies and hospitals in Turkey. Although the issue is important, a study conducted in Turkey found that almost half of the nurses reported that no institutional procedures were in place for medication safety in hospitals [26].

In this study nurses’ the of medication safety competence differ according to age, marital status, education level, professional experience, hospital type, and the types of training or courses on medication safety the nurses have received. More studies investigating nurses’ the of medication safety competence are warranted, along with studies assessing differences in the of medication safety competence between nurses with varying demographic characteristics. This study provides strong evidence for the reliability and validity of the scale in Turkish. It is also the first study to determine the medication safety competencies of nurses working in Turkey.

This study presents strong evidence that the Turkish version of the MSCS is valid and reliable among nurses. The medication safety competency levels of the nurses participating in this study were average. The assessment results of the scale provide a reference for nursing administrators to help them formulate educational plans improve the medication safety competence of nurses.

### Limitations

One of the strengths of this study is its application of the scale to a large sample. Although the methodological results are important, the adapted scale is specific to nurses in Turkey. However, the results regarding nurses’ medication safety competence obtained in the study’s second stage provide a substantial contribution to the literature.

### Electronic supplementary material

Below is the link to the electronic supplementary material.

Supplementary Material 1

## Data Availability

Data Availability StatementThe data supporting this study’s findings are available on re-quest from the corresponding author. The data are not publicly available due to privacy or ethical restrictions.
