# Supplementary material for: An analysis of the psychometric properties of the medication safety competence scale in Turkish
Source: BMC Nurs. 2024 Aug 21;23:578. doi: 10.1186/s12912-024-02240-0 (PMC11337636; doi:10.1186/s12912-024-02240-0)
Supplement: Supplementary file 1 — Supplementary Material 1 [file 12912_2024_2240_MOESM1_ESM.pdf]

Medication Safety Competence Scale for Nurses

**I feel confident about…**	**Strongly Disagree**	**Disagree**	**Neither Agree nor Disagree**	**Disagree**	**Strongly Disagree**
1. Planning care during medication process	☐	☐	☐	☐	☐
2. Evaluating the need for medication by checking patients' condition and examination results before administering medication	☐	☐	☐	☐	☐
3. Administration of medication according to the right principles (right patient, drug, dose, route and time)	☐	☐	☐	☐	☐
4. Detecting drug-related adverse effects (undesirable and harmful effects).	☐	☐	☐	☐	☐
5. Giving confidence to patients and their relatives during the medication process.	☐	☐	☐	☐	☐
6. Giving confidence to the patient by establishing clear and consistent communication	☐	☐	☐	☐	☐
7. Evaluation of my nursing practice during the medication process	☐	☐	☐	☐	☐
8. Recording the evaluation, planning, administration and evaluation of results of medication.	☐	☐	☐	☐	☐
9. Understanding the role of human factors affecting medication safety such as fatigue.	☐	☐	☐	☐	☐
10. Showing and expressing an inquiring attitude when seeing something that may be unsafe	☐	☐	☐	☐	☐
11. Defining preventive actions for medication safety	☐	☐	☐	☐	☐
12. Finding information about medication from different sources (eg drug information management system, hospital pharmacies, literature information, etc.)	☐	☐	☐	☐	☐
13. Establishing individual communication according to the condition of the patients during the drug medication process	☐	☐	☐	☐	☐

**I feel confident about…**	**Strongly Disagree**	**Disagree**	**Neither Agree nor Disagree**	**Disagree**	**Strongly Disagree**
14. Using information technology and information systems (e.g. barcodes, electronic medical records) for drug safety	□	□	□	□	□
15. Administering medication according to hospital medication management guidelines (for example, high-risk medication guidelines)	□	□	□	□	□
16. Coping promptly according to hospital protocol when adverse drug events (adverse and harmful effects) occur	□	□	□	□	□
17. Receiving regular medication safety training	□	□	□	□	□
18. Understanding the role of environmental factors influencing medication safety, such as workflow, ergonomics, and resources	□	□	□	□	□
19. Establishing preventive measures when medication errors or near misses occur	□	□	□	□	□
20. Communicating effectively with multidisciplinary members to address medication safety issues	□	□	□	□	□
21. Responding quickly to hospital protocol when medication errors or near misses occur	□	□	□	□	□
22. Seeking to create a supportive environment that encourages people to talk about problems when medication errors or near misses occur	□	□	□	□	□
23. Regularly assessing my knowledge of medication safety	□	□	□	□	□
24. Practicing medication by taking responsibility for the safety of patients	□	□	□	□	□
25. Reporting adverse medication events (adverse and harmful effects) by reporting system	□	□	□	□	□
26. Providing effective patient education to help patients talk about the symptoms of adverse (undesirable and harmful) medication effects.	□	□	□	□	□
27. Collaborating with other departments (eg pharmacy, laboratory, other service, etc.) for medication safety	□	□	□	□	□

I feel confident about…	Strongly Disagree	Disagree	Neither Agree nor Disagree	Disagree	Strongly Disagree
28. Reporting to a supervisor nurse or supervisor when medication errors or near misses occur	□	□	□	□	□
29. Improving the complexes and weaknesses of medication safety… (e.g. drug malpractice)	□	□	□	□	□
30. Sharing the decision-making process with the multidisciplinary team to address medication safety issues	□	□	□	□	□
31. Reporting medication administration errors or near misses according to the reporting system	□	□	□	□	□
32. Analyzing the case to find the root cause of medication failure	□	□	□	□	□
33. Collaborating with a multidisciplinary team to address medication safety issues	□	□	□	□	□
34. Establishing preventive measures when adverse drug events (adverse and harmful effects) occur	□	□	□	□	□
35. Identifying the root cause instead of blaming the individual when medication errors or near misses occur	□	□	□	□	□
36. Carefully administering medication as a professional	□	□	□	□	□
